# Quality Assuring the ‘Triangle of Sleep’ Psychoeducational Group Intervention for People Presenting with Neurological Conditions and Sleep Difficulties Using a Co-Production Model

**DOI:** 10.1192/bjo.2026.11484

**Published:** 2026-06-30

**Authors:** Freya Stephenson, Sarah Allester, Jessica Sidratul, Yahye Mohamud, Jake Smith

**Affiliations:** King's College London, London, United Kingdom

## Abstract

**Aims:**

Sleep difficulties affect up to 58% of individuals with Functional Neurological Disorder and other neuropsychiatric presentations. The Triangle of Sleep is a clinician-led pilot psychoeducational intervention combining learning points from Cognitive Behaviouralmodel for Insomnia (CBT-I), with behavioural principles for restructuring of day-routine and mindfulness-based exercises, designed to improve sleep. This quality improvement project worked alongside the intervention team to independently collect staff team and patient feedback using two co-production cycles to implement improvements in design and delivery of the intervention.

**Methods:**

Participants with sleep difficulties were recruited from the East Kent Neuropsychiatry Clinic to attend three one-hour group sessions covering sleep hygiene, day structuring, and mindfulness. The Plan–Do–Study–Act method was used to guide the approach. An initial scoping review identified how existing guided self-help interventions were quality assured. A questionnaire was developed using andragogy, and self-directed learning (SDL) principles to evaluate participant experiences. Verbal feedback was gathered from semi-structured interviews based on the questionnaire. The data was analysed by five reviewers and discussed with clinicians to produce a consensus document identifying feasible improvements, which were implemented over two PDSA cycles.

**Results:**

The first cycle received responses from 2 patients and 3 student observers. Key themes included clarity of learning objectives (LOs), limited discussion opportunities, repetition, optimal SDL material utilisation, and desire for an additional session. Themes were discussed with clinician facilitators resulting in a consensus document of proposed changes. Revisions included promotion of SDL materials and session restructuring to include clear LOs and discussion opportunities. An additional session was not implemented due to resource constraints.

Before the second cycle, the questionnaire was refined for sustainability of continuous improvement. The second pilot retained 8 participants, with 5 patient feedback responses. There was limited consensus between participants unlike the previous cycle. A key improvement was a 20% increase in use of SDL materials compared to the previous cycle.

**Conclusion:**

A co-production model proved effective for quality assuring a psychoeducational intervention. Iterative patient feedback enabled feasible, patient-centred improvements to acceptability and clarity. While immediate reflections on motivation in applying theories taught were mixed, the use of SDL materials suggests improved self-efficacy for long-term implementation of principles and potential for sleep improvement. The ‘Triangle of Sleep’ pilot provides a foundation for upscaling and the development of digital or AI-supported delivery models to increase reach and sustainability. In areas with less access to formal CBT-i this intervention could provide specialist insomnia treatment with lower clinician burden.

